# Sex Differences in Cue Competition Effects With a Conditioned Taste Aversion Preparation

**DOI:** 10.3389/fnbeh.2020.00107

**Published:** 2020-06-22

**Authors:** Rocio Angulo, Javier Bustamante, Vania Estades, Valeska Ramírez, Belén Jorquera

**Affiliations:** Instituto de Ciencias Sociales, Universidad de O’Higgins, Rancagua, Chile

**Keywords:** blocking, classical conditioning, overshadowing, sex differences, taste aversion

## Abstract

This study aimed to test whether male and female rats might show differences in cue competition effects in a conditioned taste aversion (CTA) model. Experiment 1 tested for sex differences in overshadowing. After conditioning of a flavored compound AB or only one simple flavor A (being A and B a solution of sugar 10% and salt 1%, counterbalanced), consumption of the A solution at test was larger in the former than in the latter case only in males. Thus, the usual effect of overshadowing was observed in males but not in females. Experiment 2 examined sex differences in blocking with the same stimuli used in Experiment 1. After conditioning of AB, the consumption of B was larger for the animals that previously received a single conditioning trial with A than for those that received unpaired presentations of A and the illness. As observed in Experiment 1, the typical blocking effect appeared only in males but not in females. The present findings thus support the hypothesis that sex dimorphism might be expressed in classical conditioning, or at least, in cue competition effects such as overshadowing and blocking with a taste aversion model.

## Introduction

In any environment, organisms encounter stimuli to which they must respond. Some of them, known as unconditioned stimuli (US), can naturally evoke unconditioned responses (UR). An US has commonly an emotional or motivational value for the organism; other stimuli, however, do not have this motivational value and are thus initially unable to elicit any response other than attentional ones. These stimuli might come to elicit responding if paired with an US, in which case they become conditioned stimuli (CS). The response that a CS elicits is consequently termed a conditioned response (CR). The learning process by which this occurs is called classical or Pavlovian conditioning (Pavlov, 1927).

Cue competition effects are several empirically relevant effects in Pavlovian conditioning, which refer to conditioning procedures where more than one stimulus is paired with the same US in each trial. Thus, the stimuli “compete” for the associative strength during the trial (e.g., Kamin, 1969; Mackintosh, 1971; Denniston et al., 1996; Pearce et al., 2006). When two stimuli are presented together during conditioning and are equally salient (e.g., stimulus A and B), it is generally observed that both become conditioned to a similar extent. However, the associative strength acquired by each stimulus is weaker than when each of them is conditioned alone. This effect is known as overshadowing (e.g., Pavlov, 1927; Revusky, 1971; Bond, 1983), and also occurs when the stimuli differ in their salience (e.g., Lindsey and Best, 1973; Mackintosh, 1976). In this case, the less salient stimulus is “overshadowed” by the more salient one, that is, the more salient stimulus acquires more associative strength compared to the less salient one. A second effect, called “blocking,” refers to a procedure where, after a single stimulus is conditioned alone, a second one is presented in compound with the first stimulus. After this manipulation, the second stimulus acquires less associative strength than the first one (i.e., it is “blocked”; e.g., Kamin, 1969; Westbrook and Brookes, 1988). Both effects are well established in the literature and have been replicated in a variety of species including humans (Vandorpe and De Houwer, 2005; Ellis, 2006; Prados, 2011; Rosas et al., 2017). Both have also been particularly relevant for research in Pavlovian conditioning, and for the development of theoretical explanations and mathematical models of learning (see e.g., Mallea et al., 2019).

One relevant question regarding these and other conditioning effects is whether males and females differ, i.e., whether or not a sex dimorphism operates for classical conditioning. Such learning effects are relevant for the etiology and treatment of several psychological diseases with cognitive-behavioral basis (e.g., Blechert et al., 2007; Laborda et al., 2012; Andreatta et al., 2015), and different prevalence for men and women (Westberg and Eriksson, 2008). Moreover, some physiological or biological variables such as stress, aging, or pharmacological effects seem to interact with sex (e.g., Leuner et al., 2004; Waddell et al., 2008, 2010; Westberg and Eriksson, 2008; Spivey et al., 2009; Maeng et al., 2010). Therefore, elucidating whether or not a sex dimorphism exists in classical conditioning effects might also be clinically relevant.

Most of the studies conducted with non-human animals have traditionally used male subjects, and while some have occasionally used females in their experiments (e.g., Mikulka et al., 1982), only very rarely have the studies offered adequate comparisons between males and females (e.g., Rodríguez et al., 2011, 2013). This is likely associated to several assumptions such as, for instance, that females might display a larger variability in the measures due to the estrous cycle (e.g., Shansky, 2019; see also Prendergast et al., 2014; Becker et al., 2016), or that sex differences might rely on estrogens, even when they might be equally affected by testosterone (e.g., Chambers and Sengstake, 1979). Regardless of the reason behind it, an inspection of the literature suggests that the studies about sex differences in classical conditioning seem to be few and inconsistent, with the findings depending on methodological issues such as, for example, the nature of the stimuli employed as CSs or USs, or the dependent variable (for a review see Dalla and Shors, 2009).

It has been observed in fear conditioning, for instance, that freezing responses appear earlier in males, suggesting faster fear conditioning in males than in females. However, active escape responses seem to appear earlier in females, suggesting the opposite pattern (e.g., Maren et al., 1994; Daviu et al., 2014; Gruene et al., 2015; Blume et al., 2017). Furthermore, the pattern of findings seem to vary according to the experimental model and the learning effect examined. For instance, sex differences in conditioning acquisition have been observed in fear conditioning (see above) or eye-blink conditioning (apparently being stronger in females; for instance see Waddell et al., 2008) but not in other models such as conditioned taste aversion (CTA; for instance see Randall-Thompson and Riley, 2003; Angulo and Arévalo-Romero, in press; Jones et al., 2006; Pittman et al., 2008; Rinker et al., 2008). Finally, within the same experimental model, specifically CTA, sex differences might be observed in some effects as extinction (e.g., Chambers and Sengstake, 1979; Sengstake and Chambers, 1979) or latent inhibition (e.g., Nofrey et al., 2008; Quinlan et al., 2010) but not in others as acquisition (e.g., Randall-Thompson and Riley, 2003; Jones et al., 2006; Pittman et al., 2008; Rinker et al., 2008). According to the evidence, thus, it seems reasonable to assume that the hypothesis of a general sex difference for certain conditioning effects will require a confirmation from studies using different experimental preparations.

Regarding sex differences specifically in overshadowing and blocking, it appears to have mainly been observed in navigation tasks, with sex differences, in particular, is expressed in the spatial learning domain (e.g., Saucier et al., 2002; Sava and Markus, 2005; Rodríguez et al., 2011, 2013; see also Chai and Jacobs, 2009; Prados, 2011). Overall, the results of these studies suggest that males perform better than females in navigation tasks, although other findings rather suggest that males and females might be using different strategies to solve spatial tasks (e.g., Roof and Stein, 1999; Rodríguez et al., 2011, 2013). For example, it has been observed, in the Morris water maze, that males performed better when the cues signaling a hidden platform are shapes instead of landmarks, while for females it was the opposite (Rodríguez et al., 2011, 2013). Rodriguez and colleagues also found evidence that, in males, shapes overshadowed landmarks, but in females, landmarks overshadowed shapes. In blocking, on the other hand, it was observed that in males only shapes blocked landmarks, but in females both cues produced blocking. This reciprocal blocking effect was also observed in males after more extensive training.

This evidence suggests that males and females might qualitatively differ in how spatial stimuli are processed. This does not necessarily mean, however, that males and females differ in the underlying learning processes involved in overshadowing and blocking. Examining this possibility would require assessing these cue competition effects in other experimental models for which spatial cues would not be relevant. This would be the case, for instance, in the CTA model (e.g., Garcia and Koelling, 1967). In CTA, the stimuli acting as CS used to be flavored solutions being the US an illness artificially induced by different substances as Lithium Chloride (Chambers and Sengstake, 1979; Sengstake and Chambers, 1979; Weinberg et al., 1982; Dacanay et al., 1984), cocaine (Busse et al., 2005; van Haaren and Hughes, 1990), and alcohol (Cailhol and Mormède, 2002). After CS-US pairings, the level of conditioned aversion acquired is established from the level of consumption of the CS, stronger conditioning being inferred from a low consumption level.

Sex differences have been observed in several conditioning effects using a CTA model, such as extinction or latent inhibition; the model has also been used to examine both the overshadowing (e.g., Bond, 1983; Kucharski and Spear, 1985; Kwok and Boakes, 2015, 2018) and the blocking effects (e.g., Gillan and Domjan, 1977; Westbrook and Brookes, 1988). Thus, this model appears to be a good candidate for assessing sex differences in overshadowing and blocking, and for providing relevant information for a better understanding of both sex dimorphism and cue competition effects.

Regarding overshadowing, it should be noted that even when the effect is commonly observed in the CTA model, interestingly sometimes potentiation is observed instead (see for example, Kwok and Boakes, 2015; Kwok and Boakes, 2018). Potentiation is the facilitation of conditioning when two stimuli are trained in compound (see e.g., Durlach and Rescorla, 1980; Kucharski and Spear, 1985; Pearce et al., 2006). Most of the studies of overshadowing and potentiation have been conducted in males. However, female rats have been used in some cases (e.g., Mikulka et al., 1982). Thus, sex is a potentially relevant variable to investigate the factors that cause potentiation instead of overshadowing.

The blocking effect (e.g., Kamin, 1969), on the other hand, has been extensively investigated due to its theoretical implications for associative learning theories. In particular, it has been a focus of debate as to whether the blocked learning of a stimulus—conditioned in compound with another stimulus previously conditioned with the same US—might be related to a lack of attention to the second, or to the fact that the US is already well predicted by the first stimulus. In this latter case, a novel stimulus would add no new information when it comes to predicting the occurrence of the US (e.g., Kamin, 1969; Rescorla and Wagner, 1972; Mackintosh, 1975; Pearce and Hall, 1980). Similarly, there has also been a discussion of the issue of whether latent inhibition might be linked to a deficit in attention to the pre-exposed stimulus after being repeatedly presented in the absence of a consequence, or whether the effect is due to a loss of stimulus associability (e.g., Rescorla and Wagner, 1972; Mackintosh, 1975; Pearce and Hall, 1980; Lubow, 1997; Escobar et al., 2002; Hall and Rodriguez, 2010). This debate has been renewed to account for the attenuation or abolition of latent inhibition observed in females (e.g., Quinlan et al., 2010). Sex differences in blocking—and latent inhibition—might, therefore, be informative at a theoretical level and are thus worthy of further research.

The main goal of the present study was to examine whether blocking and overshadowing differ across males and females, in the CTA model with which sex differences have been previously observed (Angulo and Arévalo-Romero, in press; see the “General Method” section below). Experiment 1 (Table 1) assessed sex differences in a standard overshadowing procedure where half of the subjects (groups Males-Over and Females-Over) received a conditioning trial with a compound of flavors (AB), while the other half (Males-Ctrl and Females-Ctrl) received a conditioning trial with one of its components (A); the aversion acquired by A in all groups was then measured in a test phase. An overshadowing effect would be observed if A becomes more aversive after being conditioned alone (Ctrl condition) than after being conditioned in compound with B (Over condition). Experiment 2 (Table 1), on the other hand, assessed sex differences also in a standard blocking procedure where half of the subject received a conditioning trial with a single stimulus A (groups Males-Blk and Females-Blk), while the other half received unpaired presentations of A and the illness defined as US (groups Males-Ctrl and Females-Ctrl). Then, all subjects received a conditioning trial with the AB compound; afterward, aversion acquired by each element (A and B) was measured. A blocking effect would be evidenced by greater consumption of B than A in the Blk condition along with greater consumption of B in the Blk condition compared to the control groups.

**Table 1:** Experimental designs for Experiment 1 (Overshadowing) and Experiment 2 (Blocking).

Experiment 1: Overshadowing			
Group	Conditioning	Test
Males-Over	AB+	A?	
Males-Ctrl	AB+	A?	
Females-Over	A+	A?	
Females-Ctrl	A+	A?	
**Experiment 2: Blocking**			
**Group**	**Conditioning 1**	**Conditioning 2**	**Test**
Males-Blk	A+	AB+	B? A?
Males-Ctrl	A/+	AB+	B? A?
Females-Blk	A+	AB+	B? A?
Females-Ctrl	A/+	AB+	B? A?

*A: sugar 10% solution for half of the subjects in each group and salt 1% solution for the remaining; B: sugar 10% solution for half of the subjects in each group and salt 1% solution for the remaining; +: Intraperitoneal injection of LiCl; ?: test; /: unpaired presentation*.

## General Method

### Subjects, Stimuli, and Apparatus

Subjects were 96 experimentally naïve male (48) and female (48) Sprague–Dawley rats with a mean weight of 413 g (range 332–505 g) at the beginning of each experiment. Two weeks before the beginning of the experiments rats were individually housed in cages with food and water ad libitum. The home cages were placed in a room with a constant temperature (24°C) and humidity (50%), artificially illuminated under a 12 h-dark/light cycle with the light period beginning at 8:00 am. The experimental sessions were conducted in the animals’ home cages.

The stimuli used were two simple flavored solutions, A and B, as well as a compound, AB, formed with such solutions. Stimulus A was a solution of 1% salt (commercial fine salt Lobos^®^) for half of the subjects, and a solution of 10% sugar (commercial white sugar Iansa^®^) for the other half, diluted in plain water presented at room temperature (idem with B, being this arrangement reversed or counterbalanced for the other half of the subjects). The AB compound was then a solution formed with 1% salt and 10% sugar for all animals. The flavored solutions were presented through 50-ml plastic tubes fitted with a metal spout. The US was a 10 ml/kg intraperitoneal injection of 0.15 M lithium chloride (LiCl).

### Procedure

The procedures used in the experiments were approved by the Animal Welfare Ethics Committee of the Universidad Autónoma de Chile. Males and females were randomly assigned to equivalent groups: Males-Over; Females-Over, Males-Ctrl, and Females-Ctrl (*n* = 8), in Experiment 1; and Males-Blk, Females-Blk; Males-Ctrl and Females-Ctrl (*n* = 16), in Experiment 2.

Experiments began by removing the water bottles from the home cages. In the next 4 days, all the animals received two daily 30-min drinking sessions (at 11:00 a.m. and 5:00 p.m.) in which they had free access to the liquid. These drinking periods remained constant for the rest of the experiment, with the fluid available being either water or flavored solutions according to different conditions for Experiments 1 and 2 from Day 5 on.

Overshadowing Procedure (Experiment 1). All subjects received a single conditioning trial in the morning session of Day 5. In this trial, the subjects of groups Males-Over and Females-Over received 10 ml of the AB compound, while the animals of Groups Males-Ctrl and Females-Ctrl received 10 ml of the A solution. After the 30-min drinking period bottles were removed from the home cages and all subjects received an intraperitoneal injection of LiCl. In the afternoon and the next recovery day (Day 6), all the animals had free access to water during the 30-min drinking periods. Finally, on Day 7, all animals had free access to the test solution (A) in a single consumption test during the morning session.

Blocking Procedure (Experiment 2). On Day 5 the animals of the Blocking groups (groups Males-Blk and Females-Blk) received a conditioning trial with A (half of the subjects in the morning session and the other half in the afternoon session), while the animals in the Control condition (groups Males-Ctrl and Females-Ctrl) received unpaired presentations of the A solution and the US. For half of the animals in these last two groups, the A solution was presented in the morning session and the LiCl injected in the afternoon (after the drinking period in which water was available), while this arrangement was reversed for the other half. After a recovery day, on Day 7 all the animals received a single conditioning trial with the AB compound (half in the morning session and a half on the afternoon session as in the previous conditioning trial) and plain water in the other session. Following a further recovery day, all subjects received two free consumption tests on the morning drinking sessions of the next 2 days. On one of these trials the animals received an unrestricted amount of Solution A and on the other one an unrestricted amount of Solution B. The order of presentation for Stimulus A and B was counterbalanced in each group. Thus, half of the animals in each group received the first test with A and the other half the first test with B. All the animals had then free access to water in the afternoon sessions of the test days.

In both experiments, the amount of fluid consumed during each session was calculated by weighing the tubes before and after the session and converting the difference to ml. An analysis of variance (ANOVA) was then conducted on the consumption values, with a statistical significance criterion of *p* <0.05. Size effects are presented as partial eta-squared ($\eta_{\text{p}}^{2}$; Table 3).

**Table 2:** Mean consumption in milliliters (ml) ± SEM in the first and second conditioning trials for groups Male-Blk, Male-Ctrl, Female-BLK, and Female-Ctrl, in Experiment 2.

Group	Conditioning Trial 1	Conditioning Trial 2
Males-Blk	8.94 (SEM ± 0.237)	7.34 (SEM ± 0.681)
Males-Ctrl	9.40 (SEM ± 0.179)	9.94 (SEM ± 0.244)
Females-blk	8.53 (SEM ± 0.261)	6.09 (SEM ± 0.431)
Females-Ctrl	9.22 (SEM ± 0.278)	8.81 (SEM ± 0.622)

**Table 3:** Statistical values for main effects, interactions, and simple effects analyses in the test phases of experiments 1 and 2.

Effect	*F*	*p*-value	Effect size
**Experiment 1: Overshadowing**
CS	6.36	0.018*	0.18
Sex	4.94	0.488	0.017
CS × Sex	1.17	0.282	0.04
CS (Males)	4.92	0.043*	0.26
CS (Females)	1.52	0.23	0.098
**Experiment 2: Blocking**
Stimulus	0.289	0.593	0.005
Sex	0.259	0.613	0.004
Experimental Condition	2.68	0.106	0.043
Stimulus × Sex	0.773	0.383	0.013
Stimulus × Experimental Condition	17.418	0.001*	0.225
Stimulus × Experimental Condition × Sex	9.59	0.01*	0.138
Sex × Experimental Condition	0.007	0.932	0.000
Stimulus (Males)	0.045	0.834	0.001
Experimental Condition (Males)	0.895	0.352	0.029
Stimulus × Experimental	20.4	0.001*	0.4
Condition (Males)
Stimulus (Females)	1.42	0.242	0.045
Experimental Condition (Females)	2.28	0.141	0.071
Stimulus × Experimental	0.822	0.372	0.027
Condition (Females)
Stimulus (Males-Blk)	8.46	0.011*	0.36
Stimulus (Males-Ctrl)	12.36	0.01*	0.452

Effect size indicates partial eta-squared. *Statistical significance.

## Results and Discussion

### Experiment 1: Overshadowing

The mean consumption during the conditioning trial was 8.9 (SEM ± 0.280), 9.3 (SEM ± 0.050), 8.2 (SEM ± 0.467), and 9.2 (SEM ± 0.102) ml, for the groups Males-Over, Males-Ctrl, Females-Over and Females- Ctrl, respectively. A 2 (Sex) × 2 (CS) ANOVA conducted on these data revealed an effect of CS, *F*_(1,28)_ = 5.73, *p* = 0.024, $\eta_{\text{p}}^{2}$ = 0.17, with the consumption of AB being lower than that of flavor A. Neither the effect of Sex, *F*_(1,28)_ = 1.93, *p* = 0.175, $\eta_{\text{p}}^{2}$ = 0.065, or the Sex × CS interaction, *F*_(1,28)_ = 1.03, *p* = 0.317, $\eta_{\text{p}}^{2}$ = 0.036, were significant.

**Figure 1:**
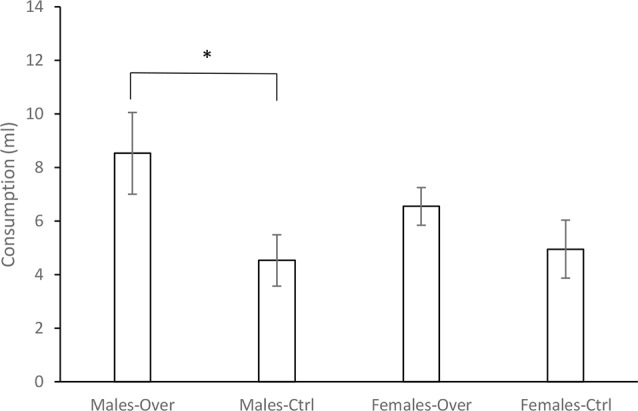
Consumption of the A solution in milliliters (ml) ± SEM for the groups Male-Over, Male-Ctrl, Female-Over and Female-Ctrl at test, in Experiment 1. *Indicates statistical significance.

Figure 1 shows the mean consumption of A during the test. Consumption of this solution appeared to be lower when it was a single CS in comparison to the AB compound, although only in males. A 2 (Sex) × 2 (CS) ANOVA conducted on these data revealed a significant effect of CS, *F*_(1,28)_ = 6.36, *p* = 0.018, $\eta_{\text{p}}^{2}$ = 0.18. Neither Sex, *F*_(1,28)_ = 4.94, *p* = 0.488, $\eta_{\text{p}}^{2}$ = 0.017, nor the Sex × CS interaction, *F*_(1,28)_ = 1.17, *p* = 0.282, $\eta_{\text{p}}^{2}$ = 0.04, were significant. However, subsequent* a priori* planned comparisons, performed to assess directly whether or not an overshadowing effect appeared in males and females, found a significant difference between the groups Males-Over and Males-Ctrl, *F*_(1,14)_ = 4.92, *p* = 0.043, $\eta_{\text{p}}^{2}$ = 0.26, but not between the Females-Over and Females-Ctrl groups, *F*_(1,14)_ = 1.52, *p* = 0.23, $\eta_{\text{p}}^{2}$ = 0.098.

Experiment 1 found a significant overshadowing effect, that is, higher consumption of the A solution after being conditioned in compound with B than when conditioned alone. However, this effect was observed only in males, but not in females. While the statistical analysis failed to find a significant interaction between Sex and the CS, the subsequent planned comparisons revealed that differences in the consumption of A after being conditioned in compound with B or conditioned alone were not reliable for females, suggesting that in the latter case it is not possible to confirm an overshadowing effect.

The difference in the consumption of A between the Males-Over and Males-Ctrl groups was likely a consequence of the greater consumption of this solution observed in the Males-Over group. When A was conditioned alone, consumption was similar in males and females, suggesting a similar rate of conditioning for A in both cases. But when the CS was the AB compound, and the test with flavor A potentially regarded as a generalization test, consumption appeared to be higher for males. In this situation, it is not possible to reject the possibility that generalization of the aversion acquired from AB to A could be stronger for females, as has been previously suggested (e.g., Angulo and Arévalo-Romero, in press; see also Pittman et al., 2008). Regardless of the mechanisms behind the effect, the findings of Experiment 1 appear to indicate an important sex difference in Pavlovian conditioning. This is of particular interest if we consider that generalization could be an important effect in the etiology and treatment of several psychological and psychiatric disorders such as, for example, post-traumatic stress disorder or phobias (e.g., Ahmed and Lovibond, 2015; Andreatta et al., 2015; Dymond et al., 2015). Thus, test sex differences in generalization with other experimental models would be an interesting goal for further research.

It might also be worth noting the fact that consumption of AB was lower than that of A on the conditioning trial. a priori, one might suppose that a compound of flavors might be more perceptively salient than a single flavor A, with the former eliciting a stronger neophobic response. The lower consumption of AB in comparison with A could thus be a consequence of this neophobia, which could hinder conditioning (but see Rosenblum et al., 1997). However, because in this case there were no sex differences in the initial consumption of AB (or A), it is not clear how this difference in consumption on the conditioning trial might have been responsible for the main finding of Experiment 1. The lack of sex differences in the initial consumption of AB and A was, to a certain extent, surprising. Angulo and Arévalo-Romero (in press) found evidence for a stronger neophobic response in females in several experiments. However, it should be noted that in those experiments the stimuli involved lemon, and possibly such a flavor could elicit a stronger neophobic response in females than salt and sugar in the concentrations used here.

### Experiment 2: Blocking

Experiment 2 as reported here was conducted in two identical replications, with the second one conducted directly after the first experiment. Data and analyses showed in the following sections correspond to both experiments taken together; a 2 (Sex) × 2 (Experimental Condition) × 2 (Replication) ANOVA conducted on data from both trials and the test showed no effect of Replication nor any interaction, all *F*s <1.17, all *p*s > 0.284.

Table 2 shows the mean consumption of the four groups on the first and second conditioning trials. On the first conditioning trial, consumption of Solution A appeared to be slightly lower for females; however, a 2 (Sex) × 2 (Experimental Condition) ANOVA showed no effect of Sex, *F*_(1,60)_ = 1.54, *p* = 0.219, $\eta_{\text{p}}^{2}$ = 0.025. There was a main effect of Condition, *F*_(1, 60)_ = 5.67, *p* = 0.02, $\eta_{\text{p}}^{2}$ =.086, but no interaction, *F* <1. On the second conditioning trial, consumption of the AB compound appeared to be lower for females, and lower for the animals that were previously conditioned relative to the control groups that received unpaired presentations of A and the US. In this case, a 2 (Sex) × 2 (Experimental Condition) ANOVA confirmed a significant effect of Sex, *F*_(1,60)_ = 5.12, *p* = 0.027, $\eta_{\text{p}}^{2}$ = 0.079, and Experimental Condition, *F*_(1,60)_ = 25.68, *p* <0.001, $\eta_{\text{p}}^{2}$ = 0.3, but no interaction, *F* <1.

**Figure 2:**
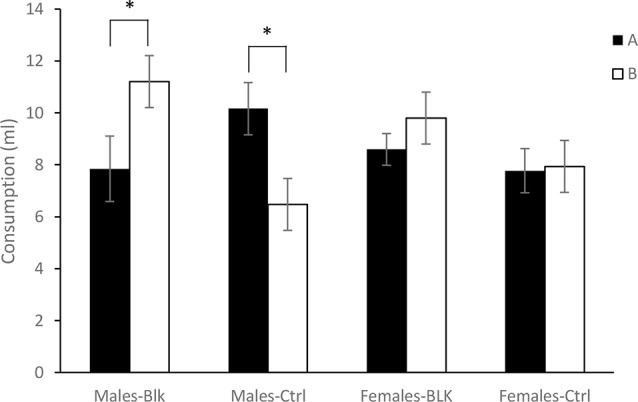
Consumption of the B solution in milliliters (ml) ± SEM for the groups Male-Blk, Male-Ctrl, Female-BLK and Female-Ctrl at test, in Experiment 2. *Indicates statistical significance.

Finally, Figure 2 shows the consumption of the solutions A and B on test for the four groups of Experiment 2. It appears that consumption of A and B was similar in females. However, in males, there was higher consumption of B than A for the Blocking group while the opposite was true for the Control group. A 2 (Sex) × 2 (Experimental Condition) × 2 (Stimulus) ANOVA conducted on these data revealed no effect of Stimulus, Sex, *F*s <1, or Condition, *F*_(1,60)_ = 2.68, *p* = 0.106, $\eta_{\text{p}}^{2}$ = 0.043, but a significant Condition × Stimulus interaction, *F*_(1,60)_ = 17.418, *p* <0.001, $\eta_{\text{p}}^{2}$ = 0.225, as well as a Sex × Condition × Stimulus interaction, *F*_(1,60)_ = 9.59, *p* <0.01, $\eta_{\text{p}}^{2}$ = 0.138. A subsequent simple effects analysis of the triple interaction found no effect of Stimulus, *F*_(1,30)_ = 1.42, *p* = 0.242, $\eta_{\text{p}}^{2}$ = 0.045, no effect of Condition, *F*_(1,30)_ = 2.28, *p* = 0.141, $\eta_{\text{p}}^{2}$ = 0.071, nor an interaction in females, *F* <1. In males on the other hand, there was no effect of Stimulus or Condition, *F*s <1, but there was a significant Condition × Stimulus interaction, *F*_(1,30)_ = 20.40, *p* <0.001, $\eta_{\text{p}}^{2}$ = 0.4. An ANOVA conducted in order to explore the Condition × Stimulus interaction found an effect of Stimulus for the Males-Blk group, *F*_(1,15)_ = 8.46, *p* = 0.011, $\eta_{\text{p}}^{2}$ = 0.36, and also for the Males-Ctrl group, *F*_(1,15)_ = 12.36, *p* <0.01, $\eta_{\text{p}}^{2}$ = 0.452. Finally, an ANOVA performed separately on the intake of solutions B and A found an effect of Condition in the consumption of B, *F*_(1,30)_ = 11.95, *p* <0.01, $\eta_{\text{p}}^{2}$ = 0.285, but not for A, *F*_(1,30)_ = 2.063, *p* = 0.161, $\eta_{\text{p}}^{2}$ = 0.064.

Experiment 2 found thus greater consumption of B than A in the Males-Blk group but not for the Females-Blk group. Moreover, the consumption of B was greater for the Males-Blk than the Males-Ctrl group but similar for the Females-Blk and Females-Ctrl groups. Therefore, a typical blocking effect was observed in males but not in females in this CTA preparation.

In males, an effect of the CS was also observed in the Control group, but in this case, this difference reflected a lower consumption of B than of A. This finding could indicate a stronger aversion for B than for A, but given that A and B were, in fact, the same stimulus (a salty or sweet solution, counterbalanced for each group), the latter finding might suggest that this could be a result of latent inhibition of A related to the unpaired presentation of A and the US before the compound conditioning. Because latent inhibition seems to be attenuated or abolished in females (e.g., Angulo and Arévalo-Romero, in press; Nofrey et al., 2008; Quinlan et al., 2010) the same finding would not be obtained in females, with consumption of A and B being similar.

## General Discussion

Two experiments in rats examined whether there is any sex difference in cue competition effects, using a CTA model. Experiment 1 examined whether there was any difference between males and females in overshadowing, while Experiment 2 was conducted in blocking. The results of Experiment 1 show that overshadowing was observed in males but not in females; blocking, on the other hand, was observed in Experiment 2 also in males, but not in females.

Previous studies have reported sex differences in certain conditioning effects such as acquisition, latent inhibition, and extinction using different preparations (e.g., fear conditioning, eye-blink conditioning, or CTA; for a review see Dalla and Shors, 2009). However, the literature has also revealed notable inconsistencies between the studies (e.g., Jones et al., 2006; Rinker et al., 2008; Kim and Spear, 2015). These inconsistencies could be related to the fact that some effects have been examined with certain conditioning models while other effects have been explored with others, being these models likely involving different types of learning. On the other hand, even when the studies shared the same type of experimental procedure, they varied regarding the stimuli employed or the responses that were recorded as dependent variables. Taken together, all of the above suggests that there is a complex core of evidence regarding a potential sex dimorphism in classical conditioning, which thus far remains unexplored. However, the current situation could be overcome by adopting a systematic approach to the study of this potential sex dimorphism by exploring different conditioning effects for each type of learning.

In this logic, the present study was conducted with the primary goal of extending the evidence regarding sex differences in a CTA model. Sex differences in neophobia, latent inhibition, extinction, and generalization were previously found by Angulo and Arévalo-Romero (in press); for a review see also Dalla and Shors (2009) using a similar preparation to the one employed here. Now, the present findings would be extending our knowledge regarding sex differences in CTA with the addition that the typical overshadowing and blocking effects found here and previously in males might be not expressed, at least in the same way, in females. Perhaps these effects are not strong enough to be detected with our experimental parameters or, similarly to what has been observed in navigation tasks (Rodríguez et al., 2011, 2013), females might process the flavor-related information in a different way to males. Further research might elucidate why the overshadowing and blocking effects were not expressed in females in the same situation in which they appeared for males, for instance by addressing the potential role of a stronger generalization between stimuli in females. Regardless of the mechanism, the present experiments showed a sex difference in these effects, and help to extend our knowledge about this topic is taken into account that sex differences in generalization, and specifically in CTA, have been proposed based on a very limited amount of evidence (e.g., Angulo and Arévalo-Romero, in press, see also Pittman et al., 2008).

On the other hand, the present findings might be also relevant at a theoretical level. Even when the typical overshadowing effect was not observed in females (Experiment 1), a potentiation effect was also not observed. Thus, sex seems not to be an important factor in determining whether overshadowing or potentiation is obtained in similar situations (see e.g., Durlach and Rescorla, 1980; Kucharski and Spear, 1985; Pearce et al., 2006). Regarding blocking, that sex differences appear for latent inhibition and blocking similarly, specifically, attenuating or vanishing the effects in females, might suggest that a similar mechanism could be underlying both effects. As it was noted in the introduction, blocking (e.g., Kamin, 1969; Rescorla and Wagner, 1972; Mackintosh, 1975; Pearce and Hall, 1980) and latent inhibition (e.g., Rescorla and Wagner, 1972; Mackintosh, 1975; Pearce and Hall, 1980; Lubow, 1997; Escobar et al., 2002; Rodriguez and Hall, 2010) both have been addressed from both associative and attentional accounts. It should be noted that the current experiments were not designed specifically to contrast such accounts. Males and females do not differ in the acquisition of CTA (see the findings relative to the conditioning of A in Experiment 1 and previous literature; e.g., Randall-Thompson and Riley, 2003; Jones et al., 2006; Pittman et al., 2008; Rinker et al., 2008), but they do differ in other effects namely latent inhibition and blocking; this might support the potential role of attentional mechanisms to a greater extent than associative ones, at least in CTA models. A general sex difference during learning of a CS-US association should lead to sex differences in acquisition, while a difference in attentional processing might not be expressed if the experimental situation does not involve cue competition or previous experience with a stimulus (e.g., latent inhibition), which might lead to a decrement in attention. This hypothesis is however purely speculative at the moment but should be addressed by further research. Although the present study does not examine it, it does open a novel approach for future studies.

Classical and operant conditioning are both learning processes that are involved in the etiology and treatment of a certain number of psychological and psychiatric disorders, as well as treatments that have a cognitive-behavioral basis (e.g., Blechert et al., 2007; Laborda et al., 2012; Andreatta et al., 2015). Thus, a better understanding of conditioning effects in women is an important information gap in this field. According to previous findings, males and females differ in perceptive, motivational, and behavioral components of learning (e.g., Beatty and Beatty, 1970; Colorado et al., 2006; Spivey et al., 2009; Andreano et al., 2013), which hinders any direct comparison between sexes on a given task. This problem is similar to that faced by comparative psychologists when they are interested in comparing different species in terms of associative learning. Thus, *a priori*, one might think that the problem could be overcome by adopting similar strategies, with one such strategy being to compare males and females on different conditioning effects rather than comparing them more broadly on their performance on different tasks, which has, until now, been usually the approach taken in the biomedical and Neuroscience fields (e.g., Leuner et al., 2004; Waddell et al., 2008, 2010; Westberg and Eriksson, 2008; Spivey et al., 2009; Maeng et al., 2010). Therefore, to elucidate whether males and females differ in terms of learning processes would be a challenging but interesting and necessary goal for both experimental and comparative psychologists. In general, the present findings have supplied novel evidence for sex dimorphism in CTA and encourage further research on sex differences in learning processes.

## Data Availability Statement

The datasets generated for this study are available on request to the corresponding author.

## Ethics Statement

The procedures used in all the experiments were approved by the Animal Welfare Ethics Committee of the Universidad Autónoma de Chile.

## Author Contributions

RA designed the study, analyzed the data and wrote the manuscript. JB analyzed the data, discussed the results, and reviewed the manuscript. VE, VR, and BJ ran the experiments.

## Conflict of Interest

The authors declare that the research was conducted in the absence of any commercial or financial relationships that could be construed as a potential conflict of interest.
